# Myocardial disturbances of intermediary metabolism in Barth syndrome

**DOI:** 10.3389/fcvm.2022.981972

**Published:** 2022-08-10

**Authors:** Amanda A. Greenwell, Seyed Amirhossein Tabatabaei Dakhili, John R. Ussher

**Affiliations:** ^1^Faculty of Pharmacy and Pharmaceutical Sciences, University of Alberta, Edmonton, AB, Canada; ^2^Women and Children's Health Research Institute, University of Alberta, Edmonton, AB, Canada

**Keywords:** Barth syndrome (BTHS), cardiomyopathy, cardiac energetics, glucose oxidation, fatty acid oxidation, ketone oxidation

## Abstract

Barth Syndrome (BTHS) is a rare X-linked mitochondrial disorder due to mutations in the gene *TAFAZZIN*, which leads to immature cardiolipin (CL) remodeling and is characterized by the development of cardiomyopathy. The immature CL remodeling in BTHS results in electron transport chain respiratory defects and destabilization of supercomplexes, thereby impairing ATP production. Thus, BTHS-related cardiomyopathy appears to share metabolic characteristics of the failing heart being an “engine out of fuel.” As CL associates with numerous mitochondrial enzymes involved in ATP production, BTHS is also characterized by several defects in intermediary energy metabolism. Herein we will describe the primary disturbances in intermediary energy metabolism relating to the heart's major fuel sources, fatty acids, carbohydrates, ketones, and amino acids. In addition, we will interrogate whether these disturbances represent potential metabolic targets for alleviating BTHS-related cardiomyopathy.

## Introduction

Cardiomyopathy is the most common clinical feature and primary driver of disease outcomes in Barth syndrome (BTHS; Online Mendelian Inheritance in Man [OMIM] 302060), a rare X-linked mitochondrial disorder first described by Dr. Peter Barth and colleagues in 1983 (1, 2). Despite the broad variability in clinical presentation, which can include features such as neutropenia, skeletal myopathy, exercise intolerance, 3-methylglutaconic aciduria, and pre-pubertal growth retardation, cardiomyopathy manifests in approximately 90% of males with BTHS (3, 4). However, the severity and specific cardiomyopathic phenotype can vary both between individuals and throughout disease progression, with no definitive genotype-phenotype correlations having yet been identified. Dilated cardiomyopathy is most common, however other forms including restrictive (5), hypertrophic, and hypertrophic-dilated cardiomyopathy have also been reported (2, 6).

**Figure 1:**
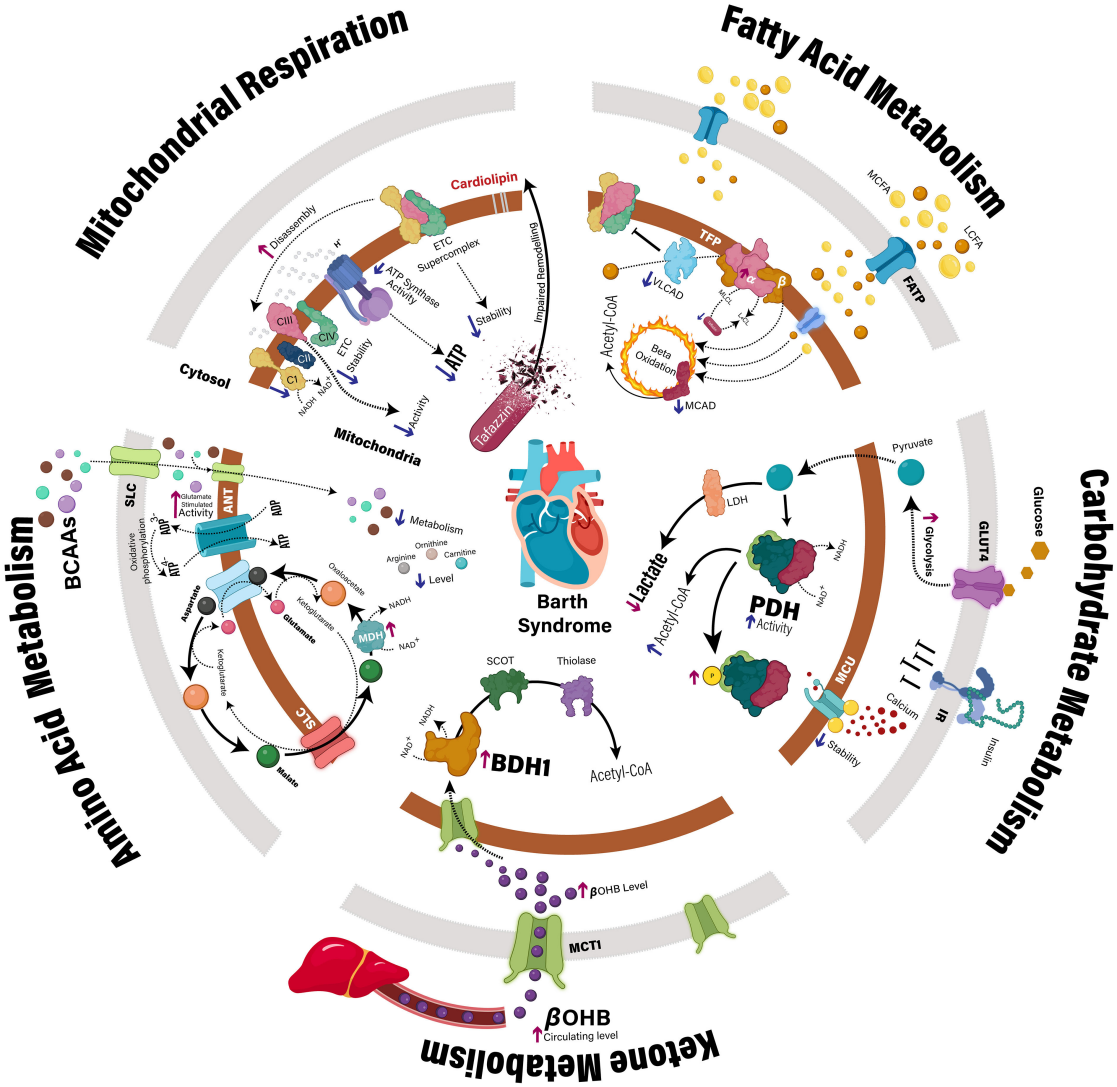
Myocardial metabolic disturbances in Barth syndrome. Clinical and preclinical studies have identified multiple mechanisms that may contribute to impaired cardiac ATP production in the context of *TAFAZZIN* deficiency. In addition to direct impairment of the mitochondrial electron transport chain, specific defects have also been identified relating to the intermediary metabolism of fatty acids, carbohydrates, ketones, and amino acids. ANT, adenine nucleotide translocase; BCAA, branched-chain amino acid, BDH1, β-hydroxybutyrate dehydrogenase; βOHB, β-hydroxybutyrate; CI-IV, complex 1–4; GLUT4, glucose transporter type 4; IR, insulin receptor; LCFA, long-chain fatty acids; LDH, lactate dehydrogenase; MCAD, medium-chain acyl-CoA dehydrogenase; MCFA, medium-chain fatty acids; MCU, mitochondrial calcium uniporter; MDH, malate dehydrogenase; P, phosphate group; PDH, pyruvate dehydrogenase; SCOT, succinyl-CoA:3-ketoacid CoA transferase; SLC, solute carrier; TFP, mitochondrial trifunctional protein; VLCAD, very long-chain acyl-CoA dehydrogenase.

BTHS is caused by mutations in the *TAFAZZIN* gene, formerly notated as *TAZ*, on chromosome Xq28, which encodes for the protein tafazzin, a phospholipid transacylase responsible for cardiolipin (CL) remodeling (3, 7). CL is a unique, dimeric, tetra-acyl phospholipid found almost exclusively in the inner mitochondrial membrane (IMM) with essential roles in the maintenance of mitochondrial morphology, and in fundamental mitochondrial functions including fission-fusion dynamics, mitophagy, apoptosis, and energy metabolism (8–10). Impaired cardiac ATP production is a principal contributor to the development and progression of heart failure (11, 12), which appears to also be present in BTHS. Indeed, the use of ^31^P magnetic resonance spectroscopy to measure the phosphocreatine to ATP ratio (PCr/ATP), an index of cardiac high-energy phosphate metabolism and energy status, was reduced in young adults (18–36 years of age) and children/adolescents with BTHS compared to healthy participants (13, 14). Nonetheless, the molecular mechanisms linking *TAFAZZIN* deficiency to perturbed myocardial energetics are presently not well-characterized. Importantly, evidence that impairments in oxidative metabolism are substrate specific in BTHS may indicate a primary defect in upstream intermediary metabolism pathways (15–17). Accordingly, the focus of this mini-review will be to characterize the alterations in intermediary metabolism that accompany *TAFAZZIN* deficiency, and to discuss the potential of targeting these pathways as a therapeutic approach to mitigate the development and progression of BTHS-related cardiomyopathy (Figure 1).

## Mitochondrial respiratory abnormalities in Barth syndrome

Notably, *TAFAZZIN* deficiency is associated with an impairment of mitochondrial respiratory capacity and ATP production, which to date, has largely been attributed to destabilization of the IMM electron transport chain (ETC) (18). Normal production of ATP in healthy mitochondria involves the oxidation of fatty acids, glucose, ketones, and amino acids to produce acetyl-CoA, which enters the Krebs cycle resulting in the generation of reducing equivalents (i.e., NADH and FADH_2_) by its 4 dehydrogenase enzymes. As these reducing equivalents deliver electrons to complex I (NADH) and II (FADH_2_) of the ETC, an electrochemical gradient across the IMM is created as electrons are passed along the ETC through a series of redox reactions, which drives the production of ATP by ATP synthase (complex V) (Figure 1).

In a *Tafazzin* deficient mouse model that relies on cre-mediated deletion of the *Tafazzin* gene containing loxP sites flanking exons 5–10, label-free quantitative proteomics revealed reductions in the relative abundances of all ETC complexes (complexes I-V) (19). Moreover, liquid chromatography (LC)-mass spectrometry (MS)/MS analysis of the cardiac proteome demonstrated a reduction in complex 1 (following normalization to citrate synthase) in left ventricular (LV) tissue from individuals with BTHS compared to non-failing control heart samples (20). Of interest, CL has been shown to be essential for the assembly of ETC supercomplexes, and supercomplexes were shown to be destabilized in hearts of *Tafazzin* knockdown (TazKD) mice secondary to doxycycline-mediated induction of a short-hairpin RNA against *Tafazzin* (21). Analysis of cardiac mitochondria isolated from cardiac-specific *Tafazzin* deficient mice prior to the development of cardiomyopathy, also revealed structural remodeling of the respiratory chain with a shift from high molecular weight supercomplexes in the ETC toward lower molecular weight forms such as heterooligomers and individual complexes (22). Furthermore, complex III activity is decreased in cardiac mitochondria isolated from TazKD mice (15, 23, 24), whereas complex V (F_1_F_0_-ATP synthase) activity is decreased in both TazKD mice and inducible pluripotent stem cell-derived cardiomyocytes from BTHS patients (15, 25). Although the detrimental consequences of *TAFAZZIN* deficiency on the structural integrity and optimal functioning of the ETC are well-defined, several studies have identified specific derangements in upstream intermediary energy metabolism pathways that we will herein interrogate (15–17). Such observations illustrate that mechanisms beyond respiratory chain dysfunction may contribute to the cardiac energy deficit in BTHS-related cardiomyopathy, while representing possible metabolic targets for intervention.

## Perturbations in myocardial fatty acid metabolism in Barth syndrome

Fatty acids are the predominant fuel source for the heart and an impaired capacity to effectively utilize fatty acids can contribute to an energy deficit and subsequent cardiac dysfunction (12, 26, 27), which appears to be present in BTHS. Myocardial fatty acid extraction and uptake following positron emission tomography (PET) imaging with [1–^11^C]palmitate were significantly reduced in young adults with BTHS compared to healthy, age-matched controls, though myocardial fatty acid β-oxidation remained similar (14). Furthermore, cardiac mitochondria isolated from TazKD mice at 2 months or 4–6 months of age, and from 2-month-old mice with a cardiac-specific *Tafazzin* deficiency demonstrated a significant repression of state 3 (ADP-stimulated) respiration rates supported by palmitoylcarnitine and malate (15, 16, 22). A decrease in palmitoylcarnitine supported state 3 respiration was also observed in permeabilized cardiac fibers with intact mitochondrial matrices from 4–6-month-old TazKD mice with preserved cardiac function, as determined by high-resolution respirometry (16). Protein expression of fatty acid binding protein and acyl-CoA synthetase, which mediate intracellular fatty acid transport and fatty acid esterification to CoA, respectively, were similar in LV tissue from male patients with BTHS and non-failing hearts (20). Furthermore, similar enzymatic activities of carnitine palmitoyltransferase I and 2 (CPT-1 and CPT-2) were observed in LV tissue homogenates from male patients with BTHS-related cardiomyopathy compared to non-failing controls (20). This suggests that BTHS-related defects in myocardial fatty acid metabolism may be downstream of mitochondrial fatty acid uptake. Supporting this premise, respiratory capacity of cardiac mitochondria from TazKD mice was decreased compared to wild-type (WT) littermates using palmitoylcarnitine, which bypasses CPT-1, whereas respiration with palmitoyl CoA plus carnitine was not reduced, providing further evidence against a specific defect in mitochondrial fatty acid uptake (16).

Alternatively, impaired tafazzin-mediated CL remodeling in BTHS may result in a selective block in long-chain fatty acid β-oxidation. Protein levels of very long-chain acyl CoA dehydrogenase (VLCAD) were decreased in LV tissue from male BTHS subjects compared to age-matched, non-failing male subjects and male subjects diagnosed with idiopathic dilated cardiomyopathy, as assessed by both immunoblotting and LC-MS/MS based proteomic profiling (20). Likewise, VLCAD protein expression was also decreased in cardiac mitochondria from 4–6-month-old TazKD mice compared to their WT littermates, as determined *via* LC-MS/MS based proteomic profiling (16). This is consistent with evidence of disrupted interactions between VLCAD and ETC supercomplexes in isolated cardiac mitochondria from 3-month-old TazKD mice (21). Of interest, protein expression of the alpha subunit of the trifunctional protein complex (TFPα) of fatty acid β-oxidation is elevated in heart tissue from BTHS subjects (20). As TFPα also possesses monolysocardiolipin (MLCL) acyltransferase activity, further investigation is required to determine whether the accumulation of MLCL in BTHS may impair the β-oxidation function of TFPα by shifting toward MLCL reacylation. Further evidence for a general defect in the enzymatic machinery of fatty acid β-oxidation in BTHS is also seen by the decline in medium-chain acyl-CoA dehydrogenase protein expression and a reduction in octanoylcarnitine-supported respiration in cardiac mitochondria from TazKD mice (16).

While the above described studies allude to impaired myocardial fatty acid β-oxidation in BTHS, there are limitations with assessing intermediary metabolism in isolated mitochondria where key cellular regulators (i.e., malonyl CoA) may be removed, and measures of protein expression do not necessarily reflect flux (28). Indeed, palmitate oxidation rates measured using [9, 10-^3^H]palmitate were elevated in the isolated working hearts of 8–10-week-old TazKD mice compared to their WT littermates (17). The reported increase in palmitate oxidation was not associated with enhanced gene or protein expression of β-oxidation enzymes and was surprisingly associated with an increase in myocardial triacylglycerol content, as well as a reduction in mRNA expression of the fatty acid transporter, cluster of differentiation 36. Reasons that may explain the elevated palmitate oxidation in the isolated working heart are unclear, though substrate oxidation rates in the working heart operate at a much higher workload than that of isolated mitochondria, and these perfusions were performed in the presence of insulin.

Despite these discrepancies in myocardial fatty acid β-oxidation in BTHS, current dogma in the field posits that stimulating myocardial fatty acid β-oxidation may be a novel approach to improve cardiac function in heart failure *via* increasing ATP production to support contraction (12). Supporting such a strategy as a therapeutic approach in BTHS, the pan-peroxisome proliferator activated receptor (PPAR) agonist, bezafibrate, is currently being investigated in the CARDIOlipin MANipulation (CARDIOMAN) trial (29). PPAR agonists, in particular PPARα, promote fatty acid β-oxidation *via* transcriptional upregulation of genes encoding for enzymes involved in fatty acid β-oxidation (30). Intriguingly, treatment of 3-month-old TazKD mice with bezafibrate for 4-months prevented the development of dilated cardiomyopathy and systolic dysfunction, which was associated with increased expression of genes involved in multiple energy metabolism pathways (31). Although the results of the CARDIOMAN trial are not yet published, a summary of the initial results of this single center, double-blinded, randomized, placebo-controlled crossover study of 11 participants with BTHS who were administered bezafibrate treatment for 15-weeks, was released by the Barth Syndrome Foundation. Assessment of cardiac function by ultrasound echocardiography revealed a significant improvement in heart strain at rest but not during peak exercise, and a trend toward improved heart chamber size. However, given that neither traditional cardiac parameters nor quality of life was changed significantly, these findings of cardiac improvement may not be clinically relevant (https://www.barthsyndrome.org/research/clinicaltrials/cardioman.html, accessed June 03, 2022). Taken together, these bezafibrate-mediated cardiac improvements in preclinical and clinical studies are encouraging and suggest that stimulating myocardial fatty acid β-oxidation may have utility in BTHS. Nonetheless, it has been suggested by others that systemic activation of PPARα with fibrates actually decreases myocardial fatty acid β-oxidation due to the stimulation of hepatic fatty acid β-oxidation and subsequent reduction in circulating lipids (32). Hence, further studies are still required to delineate whether the specific enhancement of cardiac fatty acid β-oxidation is indeed effective in alleviating BTHS-related cardiomyopathy.

## Perturbations in myocardial carbohydrate metabolism in Barth syndrome

Numerous forms of cardiovascular disease (e.g., ischemic heart disease, heart failure, diabetic cardiomyopathy) are characterized by an impairment in myocardial glucose oxidation, often due to defects in pyruvate dehydrogenase (PDH), the rate-limiting enzyme of glucose oxidation (33–35). This metabolic perturbation also appears to be present in BTHS-related cardiomyopathy, as circulating lactate levels are elevated in individuals with BTHS (36), consistent with uncoupled glucose metabolism as glycolytically-derived pyruvate is shunted to lactate vs. being oxidized *via* PDH. Further evidence for a glucose oxidation defect in BTHS has been reported in several studies utilizing the TazKD mouse model and *Tafazzin* deficient cell lines. Utilizing a XF24 Seahorse bioanalyzer, neonatal cardiac myocytes isolated from TazKD mice demonstrated an ~40% decrease in oxygen consumption coincident with an increase in extracellular acidification rates, suggestive of uncoupled glucose metabolism due to elevated glycolysis and decreased glucose oxidation (23). Furthermore, ADP-stimulated respiration in isolated cardiac subsarcolemmal and intermyofibrillar mitochondria, as well as permeabilized cardiac muscle fibers, was significantly decreased with pyruvate and malate as substrates in 4–6-month-old TazKD mice vs. their WT littermates (16). Conversely, no impairment in ADP-stimulated respiration with pyruvate as a substrate was observed in isolated cardiac mitochondria from 2-month-old TazKD mice vs. their WT littermates (15). In addition, C2C12 mouse myoblasts subjected to CRISPR/Cas9-mediated knockout of *Tafazzin* demonstrated decreased incorporation of [U-^13^C]glucose into acetyl-CoA (37). This reduction was associated with increased inhibitory phosphorylation of PDH at serine 293, resulting in an approximate 50% decrease in enzymatic activity that could be rescued by incubation with exogenous CL. Deficiency of mature CL may also blunt Ca^2+^-dependent PDH dephosphorylation by reducing the abundance and stability of the mitochondrial calcium uptake protein 1, the primary regulator of the mitochondrial calcium uniporter (MCU), thereby impairing mitochondrial Ca^2+^ uptake and PDH activation (38). A cardiac-specific downregulation of the MCU pore-forming MCUa subunit has also been observed in 10-week-old TazKD mice, which abrogates Ca^2+^ uptake in isolated cardiac mitochondria (39) and could further explain impairments in PDH activity in BTHS. This impairment in normal Ca^2+^ handling was associated with increased myofilament Ca^2+^ sensitivity and decreased cross-bridge cycling velocity, thereby contributing to diastolic dysfunction in TazKD mice and consistent with observations that a cardiac-specific deficiency of PDH promotes diastolic dysfunction (40).

BTHS-related impairments in myocardial glucose oxidation using [U-^14^C]glucose have also been observed in aerobically perfused isolated working hearts from 8–10-week-old TazKD mice, which was also associated with impaired PDH activity compared to their WT littermates (17). However, this reduction in myocardial PDH activity was independent of alterations in phosphorylation or acetylation. Of interest, the insulin-stimulated enhancement of glucose oxidation rates was also blunted in the TazKD isolated working heart suggestive of cardiac insulin resistance, which coincided with a reduction in the gene expression of glucose transporter 4. Evidence of increased and decreased circulating glucose and insulin levels, respectively, and of reduced pancreatic islet insulin secretion in TazKD mice provide further support for the paradigm of impaired insulin action in the context of *Tafazzin* deficiency (17, 41). PET imaging studies with [1-^11^C]glucose in individuals with BTHS (age 18–36 years) contrast the majority of findings in preclinical models of BTHS, as myocardial glucose extraction fraction, uptake and utilization were significantly elevated compared to healthy age-matched controls (14). However, given that myocardial glucose oxidation rates were not directly assessed in these subjects, it is plausible that the elevated myocardial glycolytic rates observed in BTHS may explain the observed increase in myocardial glucose utilization.

As the stimulation of glucose oxidation in the heart has been shown to improve cardiac function in ischemic heart disease, heart failure, and diabetic cardiomyopathy (33, 35, 42–44), this may be an exciting metabolic approach to alleviate BTHS-related cardiomyopathy. Surprisingly, treatment of 6-week-old TazKD mice for 6-weeks with dichloroacetate (DCA; 70 mM in the drinking water), a pyruvate analog that inhibits PDH kinase to prevent inhibitory phosphorylation of PDH, did not alleviate their hypertrophic cardiomyopathy phenotype despite an enhancement of PDH activity (45). Reasons that DCA may have been devoid of benefit in TazKD mice may be due to the possibility that despite PDH activity and glucose oxidation being elevated, the immature CL remodeling and destabilized ETC supercomplexes would still be present, resulting in no net improvement in myocardial ATP production. Despite the failure of DCA to alleviate hypertrophic cardiomyopathy in TazKD mice, further investigation is needed to determine whether the pharmacological optimization of glucose oxidation may still have clinical utility in BTHS. On the contrary, it is also possible that any metabolic intervention to improve myocardial oxidative metabolism in BTHS will fail unless the CL-induced defects in ETC function are also resolved.

## Perturbations in myocardial ketone metabolism in Barth syndrome

Observations that the failing heart increases its reliance on ketones as an oxidative fuel source (26, 46) has led to increased investigation of potential perturbations in myocardial ketone metabolism during the pathology of cardiovascular disease. Whether a similar shift in myocardial substrate utilization occurs in BTHS remains enigmatic, though metabolomics studies in 23 individuals with BTHS demonstrated a 1.8-fold increase in circulating β-hydroxybutyrate (βOHB) levels in comparison to 15 age-matched individuals not known to have an inborn error of metabolism (47). An increase in myocardial βOHB content was also observed in LV samples from BTHS subjects compared to age-matched non-failing control heart samples (20). Furthermore, an ~4-fold increase in myocardial protein expression of the ketone oxidation enzyme, βOHB dehydrogenase 1 (BDH1), was observed in 10-week-old TazKD mice vs. their WT littermates. Interestingly, this increased BDH1 expression did not translate into an increase in βOHB oxidation rates assessed during isolated working perfusions. As myocardial ketone oxidation rates are highly dependent on ketone delivery to the heart (48), it is plausible that identical perfusate βOHB concentrations (0.8 mM) was a limiting factor in the aforementioned study, and that ketone oxidation rates are elevated *in vivo* in TazKD mice.

It has been proposed that the salutary actions of sodium-glucose cotransporter-2 inhibitors in heart failure with reduced or preserved ejection fraction in the presence or absence of diabetes may be due in part to increasing circulating ketone levels and subsequent myocardial ketone oxidation (49–51). Accordingly, interventions that optimize cardiac ketone oxidation may represent a potential target to alleviate BTHS-related cardiomyopathy. Moreover, such a strategy may prove to be a more viable metabolic target, due to the enzymes of ketone oxidation not being as reliant on interactions with CL for their activity, as they are primarily localized to the mitochondrial matrix (52, 53).

## Perturbations in myocardial amino acid metabolism in Barth syndrome

Although amino acids contribute minimally to cardiac ATP production under normal physiological conditions (54), combined glutamate and malate-supported state 3 respiration was upregulated by 45–68% in cardiac mitochondria isolated from TazKD mice 4–6 months of age compared to their WT littermates (16). Because *TAFAZZIN* deficiency may reduce CoA availability, it was postulated that the increased reliance on glutamate metabolism manifests since glutamate can be oxidized through the malate-aspartate shuttle, which generates NADH through CoA-independent reactions. The upregulated glutamate oxidation in cardiac mitochondria from TazKD mice was associated with increased protein expression of malate dehydrogenase, a key enzyme of the malate-aspartate shuttle (55). Likewise, a 25% increase in glutamate-stimulated state 3 respiration was also reported in isolated cardiac mitochondria from 2-month-old TazKD mice (15). This particular study also reported a 6-fold increase in glutamate-stimulated activity of the CL-regulated adenine nucleotide translocase in TazKD mouse cardiac mitochondria, thus suggesting that aberrant CL remodeling may influence substrate selectivity of the adenine nucleotide translocase and overall ETC flux.

Circulating branched-chain amino acids (BCAAs) are positively associated with cardiovascular disease (26, 56), and there appears to be both transcriptional and proteomic downregulation of pathways involved in BCAA metabolism in hearts from TazKD mice at 2–3 months of age (15, 21). A decrease in cardiac BCAA metabolism, depending on where the restriction is, may lead to increased levels of myocardial BCAAs and their correspondent keto acids, the latter of which are proposed to explain how BCAA metabolism promotes cardiac insulin resistance and cardiac hypertrophy (57). Alternatively, it has been suggested that amino acid utilization is enhanced in individuals with BTHS vs. activity matched healthy control subjects, as they exhibited decreased serum levels of amino acids involved in Krebs Cycle anaplerosis, including arginine, ornithine and citrulline (58). Nonetheless, an increased reliance on amino acid metabolism to support energetic demands could potentially result in increased proteolysis of skeletal and cardiac muscle, which may contribute to the decreased lean mass and cardiac dysfunction characteristic of the BTHS phenotype (3). Supporting this perspective, a higher whole-body leucine rate of appearance per kg of fat-free mass, a measure of proteolysis, demonstrated a trend to being associated with worsened LV function as determined by a lower LV global strain in adolescents and young adults with BTHS (58). While there appears to be several disturbances in myocardial amino acid metabolism present in BTHS, further investigation will be required to determine whether such disturbances are a viable metabolic target to alleviate BTHS-related cardiomyopathy.

## Summary, conclusions & future directions

The development of multiple murine models of BTHS has greatly advanced our understanding of the metabolic perturbations present in BTHS, where it is clear that not only defects in ETC respiratory function, but also intermediary metabolism characterize this rare genetic disorder. As optimizing cardiac intermediary energy metabolism is being extensively pursued as a pharmacological strategy to improve cardiac function in heart failure, such an approach may also have clinical utility for managing BTHS-related cardiomyopathy. Unfortunately, the majority of studies that have tried to specifically optimize cardiac intermediary metabolism have yet to yield any major success. It does remain possible that any intervention that stimulates cardiac intermediary metabolism in BTHS in the absence of correcting the ETC respiratory defects, would not succeed since augmented oxidation of fuel (i.e., glucose) would not translate into increased ATP production to support cardiac function. As such, in order for metabolic therapy to yield benefit in BTHS it may need to be coupled with interventions that stabilize ETC respiratory function. Indeed, elamipretide is a cell-permeable, aromatic-cationic mitochondria-targeting tetrapeptide that localizes to the IMM, where it selectively associates with CL (59, 60) and is currently being tested in phase 2 studies for the treatment of BTHS (5). Although improvements in primary and secondary outcome measures were not observed in subjects with BTHS after 12 weeks during part 1 of the study, during the 36 week extension of part 2, exercise capacity, patient fatigue, 6-min walk test distances, and cardiac stroke volumes demonstrated improvement. Taken together, it will be imperative for future studies to assess whether elamipretide in combination with a metabolic therapy like DCA, which has been safely used in humans, may be a more effective approach to alleviate BTHS-related cardiomyopathy.

## Author contributions

All authors listed have made a substantial, direct, and intellectual contribution to the work and approved it for publication.

## Funding

This study was supported by a Project Grant from the Canadian Institutes of Health Research (CIHR) to JRU. AAG was supported by a Vanier Canada Graduate Scholarship from the CIHR. JRU is a Tier 2 Canada Research Chair (Pharmacotherapy of Energy Metabolism in Obesity).

## Conflict of interest

The authors declare that the research was conducted in the absence of any commercial or financial relationships that could be construed as a potential conflict of interest.

## Publisher's note

All claims expressed in this article are solely those of the authors and do not necessarily represent those of their affiliated organizations, or those of the publisher, the editors and the reviewers. Any product that may be evaluated in this article, or claim that may be made by its manufacturer, is not guaranteed or endorsed by the publisher.
